# 2-(3-Cyano-4-{7-[1-(2-hy­droxy­eth­yl)-3,3-dimethyl­indolin-2-yl­idene]hepta-1,3,5-trien­yl}-5,5-dimethyl-2,5-dihydro­furan-2-yl­idene)malononitrile

**DOI:** 10.1107/S1600536811042036

**Published:** 2011-10-22

**Authors:** Graeme J. Gainsford, M. Delower H. Bhuiyan, Andrew J. Kay

**Affiliations:** a Industrial Research Limited, PO Box 31-310, Lower Hutt, New Zealand 5010

## Abstract

The title compound, C_29_H_28_N_4_O_2_, excluding the hydroxyethyl and methyl groups, is slightly twisted from planarity so that the terminating indol-2-yl­idene and furan-2-yl­idene moiety planes subtend a dihedral angle of 6.27 (8)°. A small inwards fold in the polymethine atom chain is consistent with centrosymmetric dimer formation *via* O—H⋯N(cyano) hydrogen bonds. In the crystal, the mol­ecules pack in layers approximately parallel to the (10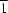) plane *via* pairs of O—H⋯N and C—H⋯N(cyano) inter­actions.

## Related literature

For general background to NLO chromophores containing an indoline donor with a 2-(3-cyano-4,5,5-trimethyl-5*H*-furan-2-yl­idene)-malontrile unit, see Gainsford *et al.* (2007 ▶, 2008 ▶, 2009 ▶). For closely related structures, see Bhuiyan *et al.* (2011 ▶). For hydrogen-motifs see: Bernstein *et al.* (1995 ▶).
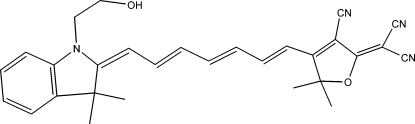


## Experimental

### 

#### Crystal data

C_29_H_28_N_4_O_2_
*M* *_r_* = 464.55
Triclinic, 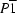

*a* = 9.3157 (4) Å
*b* = 10.5376 (4) Å
*c* = 13.4474 (6) Å
α = 101.338 (2)°
β = 100.087 (2)°
γ = 100.570 (2)°
*V* = 1241.42 (9) Å^3^
*Z* = 2
Mo *K*α radiation
μ = 0.08 mm^−1^
*T* = 124 K
0.57 × 0.38 × 0.18 mm

#### Data collection

Nonius APEXII CCD area-detector diffractometer
Absorption correction: multi-scan (*SADABS*; Bruker, 2006 ▶) *T*_min_ = 0.642, *T*_max_ = 0.746
34665 measured reflections
7739 independent reflections
5982 reflections with *I* > 2σ(*I*)
*R*_int_ = 0.034

#### Refinement

*R*[*F* ^2^ > 2σ(*F* ^2^)] = 0.046
*wR*(*F* ^2^) = 0.130
*S* = 1.02
7739 reflections
323 parameters
H atoms treated by a mixture of independent and constrained refinement
Δρ_max_ = 0.46 e Å^−3^
Δρ_min_ = −0.20 e Å^−3^

### 

Data collection: *APEX2* (Bruker, 2006 ▶); cell refinement: *SAINT* (Bruker, 2006 ▶); data reduction: *SAINT*; program(s) used to solve structure: *SHELXS97* (Sheldrick, 2008 ▶); program(s) used to refine structure: *SHELXL97* (Sheldrick, 2008 ▶); molecular graphics: *ORTEP-3* (Farrugia, 1997 ▶) and *Mercury* (Macrae *et al.*, 2006 ▶); software used to prepare material for publication: *SHELXL97*, *PLATON* (Spek, 2009) ▶ and *Mercury*.

## Supplementary Material

Crystal structure: contains datablock(s) global, I. DOI: 10.1107/S1600536811042036/im2321sup1.cif

Structure factors: contains datablock(s) I. DOI: 10.1107/S1600536811042036/im2321Isup2.hkl

Supplementary material file. DOI: 10.1107/S1600536811042036/im2321Isup3.cml

Additional supplementary materials:  crystallographic information; 3D view; checkCIF report

## Figures and Tables

**Table 1:** Hydrogen-bond geometry (Å, °)

*D*—H⋯*A*	*D*—H	H⋯*A*	*D*⋯*A*	*D*—H⋯*A*
O2—H2*O*⋯N1^i^	0.87 (2)	2.14 (2)	2.993 (2)	166.8 (16)
C26—H26*B*⋯N2^ii^	0.99	2.44	3.254 (3)	139
C29—H29*C*⋯N1^iii^	0.98	2.72	3.670 (2)	164

Symmetry codes: (i) 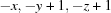; (ii) 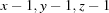; (iii) 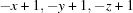.

**Fig. 1.**
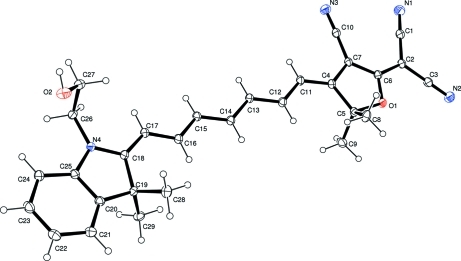
Molecular structure of the asymmetric unit (Farrugia, 1997); displacement ellipsoids are shown at the 30% probability level.

**Fig. 2.**
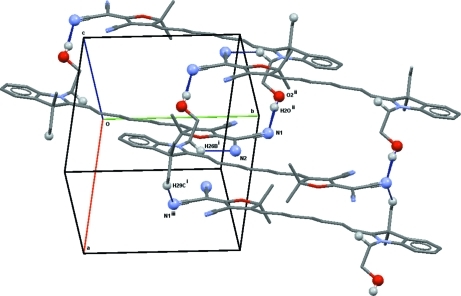
Packing diagram (Mercury, Macrae et al.,(2006)) of the unit cell showing binding interactions (dotted lines). Only hydrogen atoms involved in binding interactions are shown (all binding atoms shown as balls). Symmetry: (i) 1 + x, 1 + y, 1 + z (ii) -x, 1 - y, 1 - z (iii) 2 - x,2 - y,2 - z

## supplementary crystallographic information

### Comment

This report stems from our studies on new NLO chromophores containing an indoline donor with a well known moiety (2-(3-cyano-4,5,5-trimethyl-5*H*-furan-2-ylidene)-malontrile) (Gainsford *et al.*, 2007, 2008, 2009). It presents the structural details which were referred to in a previous paper containing the synthesis and optical properties of the title compound (6 in Bhuiyan *et al.*, 2011).

The asymmetric unit of the title compound (I) is shown in Figure 1. The furan-2-ylidene ring (C4–C7, O1) is planar while the component planar rings of the indol-2-ylidene are at 1.94 (6)° to each other similar to the 1.95 (11)° found for 2-(3-cyano-4-{5-[1-(2-hydroxy-ethyl)-3,3-dimethyl-1,3-dihydro-indol-2-ylidene] -penta-1,3-dienyl}-5,5-dimethyl-5*H*-furan-2-ylidene)-malononitrile (Bhuiyan *et al.*, 2011). The indol-2-ylidene plane (N4, C16–C23) makes an angle of 6.27 (8)° to the plane through the polymethine chain atoms (C11–C15). At this point in the polymethine chain (C15) there is a small "fold" which allows the major hydrogen bond link which binds centrosymmetrically related molecules to form a dimer (Table 1, entry 1). So whereas the dihedral angle magnitudes along the polymethine chain are close to 180 ° (176–179 °), that for C14–C15–C16–C17 is 170.58 (10)°. Thus the plane formed by the C16, C17 & C18 atoms makes an angle of 6.819 (13) ° to the preceding polymethine chain plane atoms (C4–C15) and 0.03 (11)° to the mean indoline plane. With this twist/fold combination in the polymethine chain, the indoline and furan-2-ylidene ring planes subtend 6.27 (8)°. These minor deviations from planarity appear consistent with the cell packing (noted below), the electronically delocalized planar nature of the polymethine chain and the indoline ring substituents.

The molecules are packed into layers parallel to the (1,0,-1) plane *via O*–*H*···N1(cyano), motif *R*^2^_2_(38), and *C*–*H*···N2(cyano), motif C(17) attractions (Bernstein *et al.*, 1995). The nitrogen N1 can be considered to be a bifurcated acceptor *via* a weaker supportive (methyl)C –*H*···N1(cyano) interaction (Table 1, Fig 2).

### Experimental

See details of compound 6 in Bhuiyan *et al.*(2011). Single crystals were grown by slow ether diffusion into a dichloromethane solution of the compound.

### Refinement

A total of 7 reflections within 2θ 50° were omitted as outliers (Δ(F^2^)/e.s.d. > 5.0), 1 being partially screened by the backstop.

The hydroxyl proton H_2_O was located on a difference map and refined with isotropic U(H) = 1.2*U*_eq_(O2). The methyl H atoms were constrained to an ideal geometry (C—H = 0.98 Å) with *U*_iso_(H) = 1.5*U*_eq_(C), but were allowed to rotate freely about the adjacent C—C bond. All other C-bound H atoms were placed in geometrically idealized positions and constrained to ride on their parent atoms with C—H distances of 0.95, 0.99 Å and with U(H) = 1.2*U*_eq_(C).

### Crystal data

**Table:**

C_29_H_28_N_4_O_2_	*Z* = 2
*M**_r_* = 464.55	*F*(000) = 492
Triclinic, *P*1	*D*_x_ = 1.243 Mg m^−^^3^
Hall symbol: -P 1	Mo *K*α radiation, λ = 0.71073 Å
*a* = 9.3157 (4) Å	Cell parameters from 9900 reflections
*b* = 10.5376 (4) Å	θ = 2.3–31.0°
*c* = 13.4474 (6) Å	µ = 0.08 mm^−^^1^
α = 101.338 (2)°	*T* = 124 K
β = 100.087 (2)°	Triangular, green
γ = 100.570 (2)°	0.57 × 0.38 × 0.18 mm
*V* = 1241.42 (9) Å^3^	

### Data collection

**Table:**

Nonius APEXII CCD area-detector diffractometer	7739 independent reflections
Radiation source: fine-focus sealed tube	5982 reflections with *I* > 2σ(*I*)
graphite	*R*_int_ = 0.034
Detector resolution: 8.192 pixels mm^-1^	θ_max_ = 31.0°, θ_min_ = 1.6°
φ and ω scans	*h* = −13→13
Absorption correction: multi-scan (*SADABS*; Bruker, 2006)	*k* = −15→15
*T*_min_ = 0.642, *T*_max_ = 0.746	*l* = −19→19
34665 measured reflections	

### Refinement

**Table:**

Refinement on *F*^2^	Primary atom site location: structure-invariant direct methods
Least-squares matrix: full	Secondary atom site location: difference Fourier map
*R*[*F*^2^ > 2σ(*F*^2^)] = 0.046	Hydrogen site location: inferred from neighbouring sites
*wR*(*F*^2^) = 0.130	H atoms treated by a mixture of independent and constrained refinement
*S* = 1.02	*w* = 1/[σ^2^(*F*_o_^2^) + (0.0718*P*)^2^ + 0.2125*P*] where *P* = (*F*_o_^2^ + 2*F*_c_^2^)/3
7739 reflections	(Δ/σ)_max_ = 0.001
323 parameters	Δρ_max_ = 0.46 e Å^−^^3^
0 restraints	Δρ_min_ = −0.20 e Å^−^^3^

### Special details

**Table:**

Geometry. All e.s.d.'s (except the e.s.d. in the dihedral angle between two l.s. planes) are estimated using the full covariance matrix. The cell e.s.d.'s are taken into account individually in the estimation of e.s.d.'s in distances, angles and torsion angles; correlations between e.s.d.'s in cell parameters are only used when they are defined by crystal symmetry. An approximate (isotropic) treatment of cell e.s.d.'s is used for estimating e.s.d.'s involving l.s. planes.
Refinement. Refinement of *F*^2^ against ALL reflections. The weighted *R*-factor *wR* and goodness of fit *S* are based on *F*^2^, conventional *R*-factors *R* are based on *F*, with *F* set to zero for negative *F*^2^. The threshold expression of *F*^2^ > σ(*F*^2^) is used only for calculating *R*-factors(gt) *etc*. and is not relevant to the choice of reflections for refinement. *R*-factors based on *F*^2^ are statistically about twice as large as those based on *F*, and *R*- factors based on ALL data will be even larger.

### Fractional atomic coordinates and isotropic or equivalent isotropic displacement parameters (Å^2^)

**Table:**

	*x*	*y*	*z*	*U*_iso_*/*U*_eq_	
O1	0.71795 (8)	0.82289 (7)	0.92717 (6)	0.02507 (16)	
O2	−0.35065 (10)	−0.26989 (9)	0.19623 (8)	0.0373 (2)	
H2O	−0.4383 (19)	−0.2514 (16)	0.1842 (13)	0.045*	
N1	0.66590 (15)	1.24644 (11)	0.87339 (10)	0.0448 (3)	
N2	0.93528 (13)	1.09871 (10)	1.10997 (9)	0.0392 (3)	
N3	0.45206 (12)	0.98613 (11)	0.66154 (9)	0.0366 (2)	
N4	−0.02761 (10)	−0.24219 (8)	0.24132 (7)	0.02330 (18)	
C1	0.70116 (13)	1.15797 (11)	0.90100 (9)	0.0303 (2)	
C2	0.74324 (12)	1.04986 (10)	0.93699 (9)	0.0257 (2)	
C3	0.84953 (13)	1.07704 (10)	1.03239 (9)	0.0284 (2)	
C4	0.55395 (11)	0.72345 (10)	0.76525 (8)	0.02158 (19)	
C5	0.64214 (11)	0.69063 (9)	0.85922 (8)	0.0221 (2)	
C6	0.67975 (11)	0.91863 (10)	0.88274 (8)	0.0227 (2)	
C7	0.57921 (12)	0.86372 (10)	0.78615 (8)	0.0231 (2)	
C8	0.54462 (13)	0.62225 (11)	0.92134 (9)	0.0296 (2)	
H8A	0.6078	0.6126	0.9846	0.044*	
H8B	0.4896	0.5343	0.8793	0.044*	
H8C	0.4735	0.6757	0.9403	0.044*	
C9	0.76526 (13)	0.62185 (12)	0.83557 (10)	0.0331 (3)	
H9A	0.8286	0.6736	0.7998	0.050*	
H9B	0.7210	0.5328	0.7909	0.050*	
H9C	0.8260	0.6146	0.9006	0.050*	
C10	0.51171 (12)	0.93438 (10)	0.71858 (9)	0.0260 (2)	
C11	0.46959 (12)	0.63644 (10)	0.67482 (8)	0.0242 (2)	
H11	0.4263	0.6745	0.6218	0.029*	
C12	0.44121 (11)	0.49721 (10)	0.65314 (8)	0.0236 (2)	
H12	0.4773	0.4566	0.7067	0.028*	
C13	0.36367 (11)	0.41618 (10)	0.55813 (8)	0.0236 (2)	
H13	0.3289	0.4567	0.5042	0.028*	
C14	0.33368 (11)	0.27742 (10)	0.53722 (8)	0.0231 (2)	
H14	0.3719	0.2376	0.5906	0.028*	
C15	0.25179 (11)	0.19435 (10)	0.44357 (8)	0.0237 (2)	
H15	0.2183	0.2325	0.3879	0.028*	
C16	0.21690 (11)	0.05615 (10)	0.42834 (8)	0.0239 (2)	
H16	0.2631	0.0179	0.4802	0.029*	
C17	0.11797 (12)	−0.02769 (10)	0.34110 (8)	0.0245 (2)	
H17	0.0762	0.0114	0.2883	0.029*	
C18	0.07525 (11)	−0.16581 (10)	0.32523 (8)	0.02182 (19)	
C19	0.13282 (11)	−0.25464 (10)	0.39245 (8)	0.02175 (19)	
C20	0.03818 (11)	−0.38962 (10)	0.33335 (8)	0.0231 (2)	
C21	0.02942 (13)	−0.51323 (11)	0.35538 (9)	0.0282 (2)	
H21	0.0881	−0.5230	0.4175	0.034*	
C22	−0.06712 (13)	−0.62311 (11)	0.28469 (10)	0.0310 (2)	
H22	−0.0736	−0.7088	0.2985	0.037*	
C23	−0.15338 (13)	−0.60900 (11)	0.19486 (10)	0.0313 (2)	
H23	−0.2172	−0.6856	0.1473	0.038*	
C24	−0.14916 (12)	−0.48553 (11)	0.17230 (9)	0.0291 (2)	
H24	−0.2096	−0.4754	0.1110	0.035*	
C25	−0.05204 (11)	−0.37760 (10)	0.24393 (8)	0.0236 (2)	
C26	−0.11143 (12)	−0.19416 (10)	0.15981 (8)	0.0255 (2)	
H26A	−0.1409	−0.2645	0.0946	0.031*	
H26B	−0.0462	−0.1162	0.1472	0.031*	
C27	−0.25032 (13)	−0.15534 (11)	0.18821 (10)	0.0303 (2)	
H27A	−0.2226	−0.0883	0.2553	0.036*	
H27B	−0.2992	−0.1158	0.1343	0.036*	
C28	0.10768 (13)	−0.22202 (12)	0.50358 (9)	0.0312 (2)	
H28A	0.0018	−0.2228	0.5011	0.047*	
H28B	0.1688	−0.1339	0.5407	0.047*	
H28C	0.1365	−0.2886	0.5399	0.047*	
C29	0.29911 (11)	−0.25018 (11)	0.39367 (9)	0.0274 (2)	
H29A	0.3305	−0.3181	0.4273	0.041*	
H29B	0.3593	−0.1623	0.4323	0.041*	
H29C	0.3134	−0.2673	0.3222	0.041*	

### Atomic displacement parameters (Å^2^)

**Table:**

	*U*^11^	*U*^22^	*U*^33^	*U*^12^	*U*^13^	*U*^23^
O1	0.0285 (4)	0.0173 (3)	0.0231 (4)	0.0011 (3)	−0.0028 (3)	0.0013 (3)
O2	0.0313 (4)	0.0392 (5)	0.0459 (5)	0.0108 (4)	0.0072 (4)	0.0191 (4)
N1	0.0584 (7)	0.0291 (5)	0.0429 (7)	0.0147 (5)	−0.0008 (5)	0.0053 (5)
N2	0.0450 (6)	0.0222 (4)	0.0384 (6)	0.0009 (4)	−0.0083 (5)	0.0012 (4)
N3	0.0394 (5)	0.0324 (5)	0.0355 (6)	0.0090 (4)	0.0001 (4)	0.0090 (4)
N4	0.0228 (4)	0.0189 (4)	0.0235 (4)	0.0038 (3)	−0.0027 (3)	0.0018 (3)
C1	0.0355 (6)	0.0228 (5)	0.0280 (6)	0.0067 (4)	0.0021 (4)	−0.0003 (4)
C2	0.0282 (5)	0.0187 (4)	0.0254 (5)	0.0033 (4)	0.0005 (4)	0.0005 (4)
C3	0.0322 (5)	0.0161 (4)	0.0312 (6)	0.0021 (4)	0.0012 (4)	0.0009 (4)
C4	0.0229 (4)	0.0192 (4)	0.0200 (5)	0.0020 (3)	0.0038 (4)	0.0020 (3)
C5	0.0236 (4)	0.0168 (4)	0.0208 (5)	−0.0002 (3)	0.0009 (4)	0.0007 (3)
C6	0.0240 (4)	0.0189 (4)	0.0229 (5)	0.0032 (4)	0.0037 (4)	0.0020 (4)
C7	0.0259 (5)	0.0195 (4)	0.0210 (5)	0.0037 (4)	0.0023 (4)	0.0018 (4)
C8	0.0312 (5)	0.0296 (5)	0.0231 (5)	−0.0034 (4)	0.0020 (4)	0.0074 (4)
C9	0.0277 (5)	0.0281 (5)	0.0380 (6)	0.0076 (4)	0.0015 (5)	−0.0010 (5)
C10	0.0287 (5)	0.0213 (4)	0.0244 (5)	0.0041 (4)	0.0028 (4)	0.0013 (4)
C11	0.0267 (5)	0.0213 (4)	0.0206 (5)	0.0027 (4)	0.0018 (4)	0.0015 (4)
C12	0.0235 (4)	0.0214 (4)	0.0218 (5)	0.0014 (4)	0.0017 (4)	0.0018 (4)
C13	0.0224 (4)	0.0216 (4)	0.0225 (5)	0.0032 (4)	0.0000 (4)	0.0010 (4)
C14	0.0199 (4)	0.0214 (4)	0.0238 (5)	0.0023 (3)	0.0012 (4)	0.0014 (4)
C15	0.0215 (4)	0.0211 (4)	0.0247 (5)	0.0034 (4)	0.0014 (4)	0.0011 (4)
C16	0.0215 (4)	0.0216 (4)	0.0248 (5)	0.0040 (4)	0.0011 (4)	0.0007 (4)
C17	0.0244 (5)	0.0198 (4)	0.0248 (5)	0.0035 (4)	−0.0005 (4)	0.0014 (4)
C18	0.0192 (4)	0.0207 (4)	0.0223 (5)	0.0042 (3)	0.0005 (3)	0.0014 (4)
C19	0.0195 (4)	0.0224 (4)	0.0214 (5)	0.0043 (3)	0.0008 (3)	0.0043 (4)
C20	0.0210 (4)	0.0215 (4)	0.0260 (5)	0.0052 (4)	0.0042 (4)	0.0042 (4)
C21	0.0292 (5)	0.0261 (5)	0.0316 (6)	0.0089 (4)	0.0064 (4)	0.0095 (4)
C22	0.0326 (5)	0.0218 (5)	0.0400 (6)	0.0063 (4)	0.0111 (5)	0.0077 (4)
C23	0.0280 (5)	0.0210 (5)	0.0394 (6)	0.0022 (4)	0.0045 (5)	0.0003 (4)
C24	0.0265 (5)	0.0226 (5)	0.0317 (6)	0.0035 (4)	−0.0018 (4)	0.0005 (4)
C25	0.0218 (4)	0.0187 (4)	0.0275 (5)	0.0041 (4)	0.0019 (4)	0.0028 (4)
C26	0.0273 (5)	0.0232 (5)	0.0223 (5)	0.0050 (4)	−0.0025 (4)	0.0046 (4)
C27	0.0292 (5)	0.0281 (5)	0.0315 (6)	0.0090 (4)	−0.0008 (4)	0.0069 (4)
C28	0.0317 (5)	0.0339 (6)	0.0253 (5)	0.0035 (4)	0.0070 (4)	0.0040 (4)
C29	0.0205 (4)	0.0302 (5)	0.0316 (6)	0.0067 (4)	0.0033 (4)	0.0085 (4)

### Geometric parameters (Å, °)

**Table:**

O1—C6	1.3400 (13)	C14—H14	0.9500
O1—C5	1.4793 (12)	C15—C16	1.3980 (14)
O2—C27	1.4194 (14)	C15—H15	0.9500
O2—H2O	0.869 (17)	C16—C17	1.3861 (14)
N1—C1	1.1491 (16)	C16—H16	0.9500
N2—C3	1.1515 (16)	C17—C18	1.3993 (14)
N3—C10	1.1493 (15)	C17—H17	0.9500
N4—C18	1.3492 (13)	C18—C19	1.5258 (14)
N4—C25	1.4115 (13)	C19—C20	1.5106 (14)
N4—C26	1.4596 (13)	C19—C28	1.5351 (15)
C1—C2	1.4142 (16)	C19—C29	1.5383 (14)
C2—C6	1.3952 (14)	C20—C21	1.3829 (14)
C2—C3	1.4195 (16)	C20—C25	1.3831 (15)
C4—C11	1.3783 (14)	C21—C22	1.3924 (16)
C4—C7	1.4160 (14)	C21—H21	0.9500
C4—C5	1.5178 (14)	C22—C23	1.3790 (18)
C5—C9	1.5114 (16)	C22—H22	0.9500
C5—C8	1.5182 (14)	C23—C24	1.3881 (16)
C6—C7	1.4066 (14)	C23—H23	0.9500
C7—C10	1.4189 (15)	C24—C25	1.3880 (14)
C8—H8A	0.9800	C24—H24	0.9500
C8—H8B	0.9800	C26—C27	1.5142 (16)
C8—H8C	0.9800	C26—H26A	0.9900
C9—H9A	0.9800	C26—H26B	0.9900
C9—H9B	0.9800	C27—H27A	0.9900
C9—H9C	0.9800	C27—H27B	0.9900
C11—C12	1.4036 (14)	C28—H28A	0.9800
C11—H11	0.9500	C28—H28B	0.9800
C12—C13	1.3801 (14)	C28—H28C	0.9800
C12—H12	0.9500	C29—H29A	0.9800
C13—C14	1.3986 (14)	C29—H29B	0.9800
C13—H13	0.9500	C29—H29C	0.9800
C14—C15	1.3854 (14)		
			
C6—O1—C5	110.13 (8)	C15—C16—H16	118.5
C27—O2—H2O	104.6 (11)	C16—C17—C18	124.68 (10)
C18—N4—C25	111.32 (9)	C16—C17—H17	117.7
C18—N4—C26	125.79 (9)	C18—C17—H17	117.7
C25—N4—C26	122.80 (8)	N4—C18—C17	122.27 (10)
N1—C1—C2	178.71 (14)	N4—C18—C19	109.07 (8)
C6—C2—C1	121.63 (10)	C17—C18—C19	128.64 (9)
C6—C2—C3	119.86 (10)	C20—C19—C18	101.24 (8)
C1—C2—C3	118.48 (9)	C20—C19—C28	110.34 (8)
N2—C3—C2	179.68 (14)	C18—C19—C28	113.67 (9)
C11—C4—C7	125.61 (10)	C20—C19—C29	110.24 (9)
C11—C4—C5	127.83 (9)	C18—C19—C29	110.21 (8)
C7—C4—C5	106.52 (8)	C28—C19—C29	110.77 (9)
O1—C5—C9	106.02 (8)	C21—C20—C25	119.45 (10)
O1—C5—C4	103.28 (7)	C21—C20—C19	131.01 (10)
C9—C5—C4	113.74 (9)	C25—C20—C19	109.53 (9)
O1—C5—C8	105.87 (8)	C20—C21—C22	118.71 (11)
C9—C5—C8	113.07 (9)	C20—C21—H21	120.6
C4—C5—C8	113.70 (9)	C22—C21—H21	120.6
O1—C6—C2	117.20 (9)	C23—C22—C21	120.74 (10)
O1—C6—C7	110.88 (8)	C23—C22—H22	119.6
C2—C6—C7	131.91 (10)	C21—C22—H22	119.6
C6—C7—C4	109.13 (9)	C22—C23—C24	121.58 (10)
C6—C7—C10	126.75 (9)	C22—C23—H23	119.2
C4—C7—C10	124.12 (9)	C24—C23—H23	119.2
C5—C8—H8A	109.5	C25—C24—C23	116.57 (11)
C5—C8—H8B	109.5	C25—C24—H24	121.7
H8A—C8—H8B	109.5	C23—C24—H24	121.7
C5—C8—H8C	109.5	C20—C25—C24	122.91 (10)
H8A—C8—H8C	109.5	C20—C25—N4	108.74 (9)
H8B—C8—H8C	109.5	C24—C25—N4	128.34 (10)
C5—C9—H9A	109.5	N4—C26—C27	112.10 (9)
C5—C9—H9B	109.5	N4—C26—H26A	109.2
H9A—C9—H9B	109.5	C27—C26—H26A	109.2
C5—C9—H9C	109.5	N4—C26—H26B	109.2
H9A—C9—H9C	109.5	C27—C26—H26B	109.2
H9B—C9—H9C	109.5	H26A—C26—H26B	107.9
N3—C10—C7	176.75 (12)	O2—C27—C26	109.27 (9)
C4—C11—C12	126.54 (10)	O2—C27—H27A	109.8
C4—C11—H11	116.7	C26—C27—H27A	109.8
C12—C11—H11	116.7	O2—C27—H27B	109.8
C13—C12—C11	123.42 (10)	C26—C27—H27B	109.8
C13—C12—H12	118.3	H27A—C27—H27B	108.3
C11—C12—H12	118.3	C19—C28—H28A	109.5
C12—C13—C14	123.30 (10)	C19—C28—H28B	109.5
C12—C13—H13	118.4	H28A—C28—H28B	109.5
C14—C13—H13	118.4	C19—C28—H28C	109.5
C15—C14—C13	124.16 (10)	H28A—C28—H28C	109.5
C15—C14—H14	117.9	H28B—C28—H28C	109.5
C13—C14—H14	117.9	C19—C29—H29A	109.5
C14—C15—C16	122.12 (10)	C19—C29—H29B	109.5
C14—C15—H15	118.9	H29A—C29—H29B	109.5
C16—C15—H15	118.9	C19—C29—H29C	109.5
C17—C16—C15	123.03 (10)	H29A—C29—H29C	109.5
C17—C16—H16	118.5	H29B—C29—H29C	109.5
			
C6—O1—C5—C9	120.90 (9)	C26—N4—C18—C19	178.39 (9)
C6—O1—C5—C4	1.02 (10)	C16—C17—C18—N4	176.41 (10)
C6—O1—C5—C8	−118.74 (9)	C16—C17—C18—C19	−4.91 (18)
C11—C4—C5—O1	175.76 (10)	N4—C18—C19—C20	−2.72 (11)
C7—C4—C5—O1	−2.02 (10)	C17—C18—C19—C20	178.46 (10)
C11—C4—C5—C9	61.33 (14)	N4—C18—C19—C28	−121.01 (10)
C7—C4—C5—C9	−116.45 (10)	C17—C18—C19—C28	60.16 (14)
C11—C4—C5—C8	−70.00 (14)	N4—C18—C19—C29	113.96 (10)
C7—C4—C5—C8	112.22 (10)	C17—C18—C19—C29	−64.86 (14)
C5—O1—C6—C2	−179.48 (9)	C18—C19—C20—C21	−176.50 (11)
C5—O1—C6—C7	0.39 (11)	C28—C19—C20—C21	−55.82 (15)
C1—C2—C6—O1	−174.82 (10)	C29—C19—C20—C21	66.84 (14)
C3—C2—C6—O1	3.24 (15)	C18—C19—C20—C25	2.87 (11)
C1—C2—C6—C7	5.35 (19)	C28—C19—C20—C25	123.54 (10)
C3—C2—C6—C7	−176.59 (11)	C29—C19—C20—C25	−113.79 (10)
O1—C6—C7—C4	−1.78 (12)	C25—C20—C21—C22	2.05 (16)
C2—C6—C7—C4	178.06 (11)	C19—C20—C21—C22	−178.64 (10)
O1—C6—C7—C10	178.17 (10)	C20—C21—C22—C23	−0.56 (17)
C2—C6—C7—C10	−2.00 (19)	C21—C22—C23—C24	−1.00 (18)
C11—C4—C7—C6	−175.50 (10)	C22—C23—C24—C25	0.98 (17)
C5—C4—C7—C6	2.34 (11)	C21—C20—C25—C24	−2.12 (16)
C11—C4—C7—C10	4.55 (17)	C19—C20—C25—C24	178.43 (10)
C5—C4—C7—C10	−177.60 (10)	C21—C20—C25—N4	177.37 (9)
C7—C4—C11—C12	−178.93 (10)	C19—C20—C25—N4	−2.08 (12)
C5—C4—C11—C12	3.69 (18)	C23—C24—C25—C20	0.58 (17)
C4—C11—C12—C13	−175.59 (10)	C23—C24—C25—N4	−178.81 (11)
C11—C12—C13—C14	−179.04 (10)	C18—N4—C25—C20	0.23 (12)
C12—C13—C14—C15	177.74 (10)	C26—N4—C25—C20	−176.59 (9)
C13—C14—C15—C16	−176.01 (10)	C18—N4—C25—C24	179.69 (11)
C14—C15—C16—C17	170.58 (10)	C26—N4—C25—C24	2.87 (17)
C15—C16—C17—C18	−177.05 (10)	C18—N4—C26—C27	−85.16 (12)
C25—N4—C18—C17	−179.40 (10)	C25—N4—C26—C27	91.18 (12)
C26—N4—C18—C17	−2.69 (16)	N4—C26—C27—O2	−64.57 (12)
C25—N4—C18—C19	1.69 (12)		

### Hydrogen-bond geometry (Å, °)

**Table:**

*D*—H···*A*	*D*—H	H···*A*	*D*···*A*	*D*—H···*A*
O2—H2O···N1^i^	0.87 (2)	2.14 (2)	2.993 (2)	166.8 (16)
C26—H26B···N2^ii^	0.99	2.44	3.254 (3)	139
C29—H29C···N1^iii^	0.98	2.72	3.670 (2)	164

Symmetry codes: (i) −*x*, −*y*+1, −*z*+1; (ii) *x*−1, *y*−1, *z*−1; (iii) −*x*+1, −*y*+1, −*z*+1.
